# Clinical Characteristics of Migraine and Their Association With the Retinal Nerve Fiber Layer Thickness

**DOI:** 10.7759/cureus.60909

**Published:** 2024-05-23

**Authors:** Ruchi Shukla, Ashutosh K Mishra, Mukesh Shukla, Archana Verma, Pragati Garg, Suyash Singh, Rajwinder Kaur, Nilakshi Banerjee

**Affiliations:** 1 Ophthalmology, All India Institute of Medical Sciences, Rae Bareli, IND; 2 Neurology, All India Institute of Medical Sciences, Rae Bareli, IND; 3 Community and Family Medicine, All India Institute of Medical Sciences, Rae Bareli, IND; 4 Neurological Surgery, All India Institute of Medical Sciences, Rae Bareli, IND

**Keywords:** headache, rnfl thickness, aura, optical coherence topography, migraine

## Abstract

Background: Migraine is characterized by recurrent episodes of unilateral, pulsating headaches. At the cerebral and ocular levels, it is recognized that the vascular narrowing and loss of blood flow are transient; however, the chronic nature of migraine may result in long-term functional and structural changes in these structures. It could result in axonal loss and an alteration in the thickness of the retinal nerve fiber layers (RNFL). This study aimed to measure the RNFL thickness, which provides a useful indication of the state of the axons and the loss of ganglion cells in migraine patients, and to find out if RNFL thickness and the clinical features of migraine are correlated.

Materials and methods: Sixty patients with migraine and 60 age-gender-matched controls were recruited. A complete neurological and ophthalmological examination was performed, and spectral-domain optical coherence tomography (SD-OCT) was done to measure RNFL.

Results: All quadrants of the retina on both sides showed non-statistically significant differences in RNFL thickness between migraine patients and controls (p-value >0.05). Furthermore, in all retinal quadrants on both sides, there was no statistically significant difference in RNFL thickness between migraine patients with aura and those without aura (p-value >0.05). Significant correlations were found between the duration of migraine disease and the superior RNFL thickness of both eyes, as well as the inferior RNFL in the right eye. There was also a significant correlation between the headache attack duration and RNFL thickness of the superior retina (p<0.05),

Conclusion: Our key finding was that when comparing migraine patients to controls, RNFL thickness did not significantly change; however, the duration of migraine disease did significantly affect RNFL thickness.

## Introduction

Migraine is a disorder characterized by recurrent headaches along with other symptoms of the nervous system, gastrointestinal tract, and autonomic nervous system [1]. Migraine headaches are classified into two syndromes: those with and those without aura. The symptoms of aura can manifest just before or during the onset of a migraine attack, or they might occur even without a headache. It is believed that migraine with aura affects approximately 10%-30% of all migraine patients [2]. The aura takes five to 20 minutes to form and persists for no more than one hour. The symptoms of aura are mainly visual in nature, including hallucinations or illusions of intense flashing lights and transient blindness.

According to Global Burden of Disease (GBD) 2019 data, migraines continue to rank second globally and first among young women as causes of disability [3]. Due to missed work or school days, decreased productivity, and the high expense of prescription drugs, migraine headaches have a negative influence on quality of life. Patients with migraines were shown to have severely impaired quality of life, leading to considerable disability [4]. It was found that patients experiencing frequent episodes have psychological, social, intellectual, and occupational effects. Both physical and psychological repercussions are possible during or after a migraine episode, and the recurring attacks lead to functional limitations [5]. The Migraine Disability Assessment Scale (MIDAS) is a tool that is useful in the comprehensive assessment and management of migraines [6]. Few data exist regarding the disability experienced by migraine patients in the Indian community.

Regarding the pathophysiology of migraine, several neurovascular, vascular, hormonal, cellular, hypoxic, and hereditary theories have been discussed. The pathogenesis of migraine involves episodic sensitization and activation of the trigeminal vascular bundle [7], which is also present in extracranial regions such as the choroid and retina. Its activation results in inflammation as well as vasodilation, followed by vasoconstriction in both the cranial and extracranial regions. At the cerebral and ocular levels, it is recognized that the vascular narrowing and loss of blood flow are transient; however, the chronic nature of migraine may result in long-term functional and structural changes in these structures [8].

The retinal nerve fiber layer (RNFL) is comparable to the brain's gray matter since the retina is said to be an embryological extension of the brain. The axonal injury in the retina can lead to a variation in the thickness of the RNFL. Retinal, choroid, and optic nerve measurements may be used to determine the effects of recurrent episodes of cerebral vascular fluctuations on the eyes. Examining the thickness of ocular structures in migraine patients is crucial because it can help detect structural changes in the eye early on before symptoms arise and permanent vision loss takes place.

Since the retina is a window to the brain, neuroscientists are interested in studying it. Over the past few years, spectral domain OCT (SD-OCT) has been used in numerous studies that have provided scientific proof of RNFL and ganglion cell layer (GCL) alterations in a variety of neurological disorders. These studies' findings suggest a relationship between RNFL thickness, brain atrophy, and clinically manifested visual loss, making the eye a useful model for researching neurodegenerative illnesses such as Parkinson's disease, multiple sclerosis, and Alzheimer's disease [9].

Recent technological advancements have made it possible to acquire high-resolution images of the optic disc, RNFL, ganglion cell, and choroid layers using new-generation SD-OCT instruments that use improved scanning speed and specialized software techniques. These instruments can perform non-invasive and reproducible evaluations and have been used clinically as an imaging method for the estimation of the thickness of these structures in a variety of neuro-ophthalmologic diseases.

The duration of the illness, its severity, and the frequency of attacks may all have an impact on alterations in the thickness of the RNFL in migraineurs. There are differences in the outcomes of the research articles that discuss RNFL thickness changes in migraine patients. Even if there is a statistically significant decrease in RNFL thickness, the involved quadrants may differ in various studies.

Our study aims to quantify the RNFL thickness in migraine patients compared to age- and gender-matched controls, ascertain whether RNFL thickness differs between migraine patients with or without aura, and demonstrate whether these structural changes are correlated with migraine features such as length of disease, frequency of migraine, duration of headache episode, and disability as assessed by MIDAS.

## Materials and methods

In this study, we recruited 60 migraine patients and 60 age- and gender-matched controls in the period between July 2023 and March 2024 from the outpatient Department of Neurology of the All India Institute of Medical Sciences, Rae Bareli, India.

Participants in our study were those patients who satisfied the International Classification of Headache Disorders-III (ICHD-III) diagnostic criteria for episodic or chronic migraine headaches [10]. These migraine patients were in the age group of 18-50 years. Excluded from the study were patients with secondary headaches, structural brain injuries, central nervous system disorders, ocular diseases such as glaucoma, cataract, optic disc edema, or retinal pathology, and pregnant women.

The control group consisted of 60 gender- and age-matched individuals who had never experienced a migraine attack and had no prior history of neurological or ocular disorders.

A comprehensive neurological examination and a semi-structured interview with a neurologist were conducted for each patient to thoroughly assess their headaches. The demographic and clinical parameters of the patients were recorded, including age, gender, and migraine-related variables including aura, duration of migraine episodes, frequency of headache attacks per month, and length of migraine disease.

The MIDAS, a questionnaire designed to evaluate headache-related impairment, was used to gauge the severity of headaches [11].

A comprehensive eye examination was performed, which included best-corrected visual acuity using Snellen’s chart, measurement of central corneal thickness, intraocular pressure using Goldmann applanation tonometry, slit lamp biomicroscopy, and fundus examination with 90D lens. The thickness of the RNFL was measured using SD-OCT (Cirrus 5000, ZEISS Medical Technology, Oberkochen, Germany).

High-resolution cross-sectional images of the retina are produced by SD-OCT. It was used with the standard RNFL thickness scan procedure. Tropicamide 1% eye drops were utilized to dilate the pupil in order to get high-resolution OCT images. The thickness of the superior, inferior, nasal, temporal, and average RNFL were assessed in all patients and controls.

Statistical methods

Microsoft Excel (Microsoft Corporation, Redmond, WA) was used to examine the data. In the case of migraine patients and controls, the quantitatively normally distributed data were displayed as mean and standard deviation (SD), while the quantitatively non-normally distributed data were shown as median and interquartile range (IQR). In regularly distributed quantitative data, the comparison between migraine patients and control groups was performed using the independent sample t-test; in non-normally distributed quantitative data, the comparison was made using the Mann-Whitney test. In order to compare migraine patients and control groups in categorical data, the chi-square test was employed. Frequency of migraine, length of headache episode, duration of migraine, MIDAS, and RNFL thickness were all correlated using Spearman's rank correlation coefficient (r). A significance threshold of p <0.05 was applied. Data were analyzed, and correlation coefficients were estimated for independent and dependent variables such as duration of migraine and RNFL thickness individually for all migraine patients. Although the subgroup analysis was done, the correlation coefficient with the respective p-values was calculated for each quadrant of RNFL thickness individually.

## Results

In this study, 60 migraine patients and 60 controls were included. There was no statistically significant difference between the two groups (p = 0.31, 0.34, respectively) with respect to age and gender. Table 1 illustrates the demographic and clinical characteristics of migraine patients and controls.

**Table 1:** Demographic profile and clinical characteristics of migraine patients compared to controls *Man-Whitney U test; #Chi-square; p-value<0.05 is considered statistically significant IQR: interquartile range; IOP: intraocular pressure

Characteristics	Migraine (n=60)	Control (n=60)	p-value
Mean age in years (median, IQR)	30.30±9.52 (29, IQR 18-46)	32.12 ± 10.20 (30, IQR 19-48)	0.31*
Gender			
Male (n=43)	19 (31.7)	24 (40.0)	0.34^#^
Female (n=77)	41 (68.3)	36 (60.0)	
Vision			
Right	0.03±0.10	0.00 ± 0.00	0.002*
Left	0.04±0.1	0.00 ± 0.00	0.002*
IOP			
Right	16.64±3.74	15.97±2.90	0.20*
Left	16.27±3.22	16.00±2.87	0.71*
Migraine with aura	17 (28.33)	---	NA
Duration of migraine (median, IQR)	3.44±3.22 (2, 0.5-10)	---	NA
Headache duration in hours (median, IQR)	6.68±2.9 (5, 4-12)	--	NA
Headache severity (MIDAS) (Median, IQR)	13.7±6.08 (14.5,3-24)	--	NA
Type of migraine			
Episodic	49 (81.7)	--	NA
Chronic	11 (18.3)	---	NA
Frequency of attack per month (median, IQR)	10, IQR 3-20	---	NA

Average and RNFL thickness in all quadrants in both right and left eyes did not show any significant difference between the RNFL thickness in migraine patients compared to controls (p-value >0.05; Table 2).

**Table 2:** The RNFL in the four quadrants of the right and left eyes in migraine patients compared to controls *Man-Whitney U test; p-value <0.05 is considered statistically significant RNFL: retinal nerve fiber layers; IQR: interquartile range

RNFL thickness	Migraine (n=60)	Control (n=60)	*p-value
Superior retina			
Right (median (IQR))	120 (84-154)	117 (97-145)	0.13
Left (median (IQR))	121 (83-146)	117 (98-143)	0.42
Inferior retina			
Right (median (IQR))	121 (75-152)	121 (100-153)	0.74
Left (median (IQR))	123 (89-151)	124 (94-148)	0.77
Nasal retina			
Right (median (IQR))	72 (40-96)	75 (63-95)	0.12
Left (median (IQR))	71 (56-93)	72 (56-91)	0.54
Temporal retina			
Right (median (IQR))	62 (49-85)	62 (48-77)	0.38
Left (median (IQR))	61 (50-80)	60 (48-76)	0.52
Average			
Right (median (IQR))	94 (60-111)	93 (77-112)	0.77
Left (median (IQR))	92 (77-110)	93 (77-110)	0.94

Migraine patients who had aura did not have a significant difference from those who did not have aura in terms of RNFL thickness in either of the retinal quadrants on either side (p-value >0.05; Table 3).

**Table 3:** Comparison of RNFL thickness in patients of migraine with and without aura (N=60) *Man-Whitney U test; p-value <0.05 is considered statistically significant RNFL: retinal nerve fiber layers; IQR: interquartile range

RNFL thickness	With aura (n=17)	Without aura (n=43)	*p-value
Superior retina			
Right (median (IQR))	123 (109-145)	119 (93-143)	0.23
Left (median (IQR))	123 (111-143)	121 (84-146)	0.66
Inferior retina			
Right (median (IQR))	119 (110-142)	121 (95-150)	0.79
Left (median (IQR))	124 (110-130)	121 (98-151)	0.79
Nasal retina			
Right (median (IQR))	73 (65-78)	72 (40-95)	0.93
Left (median (IQR))	73 (63-80)	71 (56-93)	0.41
Temporal retina			
Right (median (IQR))	60 (56-64)	63 (52-80)	0.22
Left (median (IQR))	61 (52-75)	62 (56-65)	0.87
Average			
Right (median (IQR))	95 (88-107)	94 (74-111)	0.67
Left (median (IQR))	95 (89-103)	91 (79-110)	0.38

Furthermore, episodic and chronic migraine patients did not have any significant difference in RNFL thickness (p-value >0.05; Table 4).

**Table 4:** RNFL thickness in patients with episodic migraine versus chronic migraine (N=60) *Man-Whitney U test; p-value <0.05 is considered statistically significant RNFL: retinal nerve fiber layers; IQR: interquartile range

RNFL thickness	Episodic migraine (n=49)	Chronic migraine (n=11)		*p-value
Superior retina				
Right (median (IQR))	121 (89-146)	115 (112-139)	0.83	
Left (median (IQR))	125 (95-146)	110 (98-123)	0.12	
Inferior retina				
Right (median (IQR))	121 (95-152)	115 (111-136)	0.75	
Left (median (IQR))	123 (98-151)	123 (113-126)	0.91	
Nasal retina				
Right (median (IQR))	73 (40-96)	72 (67-73)	0.52	
Left (median (IQR))	72 (56-93)	68 (62-71)	0.14	
Temporal retina				
Right (median (IQR))	63 (49-80)	62 (58-63)	0.46	
Left (median (IQR))	62 (52-80)	58 (56-66)	0.47	
Average				
Right (median (IQR))	95 (60-111)	93 (90-102)	0.97	
Left (median (IQR))	94 (80-110)	91 (86-103)	0.64	

Disability caused by migraine, as assessed by MIDAS, had no significant association with RNFL thickness in all retinal quadrants in both right and left eyes (Table 5).

**Table 5:** Correlation between MIDAS and RNFL thickness in patients with migraine (N=60) *Spearman’s correlation (ρ); p-value < 0.05 is considered statistically significant MIDAS: Migraine Disability Assessment Scale; RNFL: retinal nerve fiber layers; IQR: interquartile range

MIDAS	(r) Coef.	*p-value
Superior retina		
Right (median (IQR))	-0.10	0.14
Left (median (IQR))	-0.07	0.32
Inferior retina		
Right (median (IQR))	-0.80	0.36
Left (median (IQR))	0.03	0.79
Nasal retina		
Right (median (IQR))	-0.10	0.24
Left (median (IQR))	-0.02	0.91
Temporal retina		
Right (median (IQR))	-0.04	0.64
Left (median (IQR))	-0.14	0.22
Average		
Right (median (IQR))	0.39	0.07
Left (median (IQR))	0.01	0.97

The correlations between the RNFL thicknesses of both eyes and migraine headache characteristics are shown in Table 6. Significant correlations were found between the duration of migraine disease and the superior RNFL thickness of both eyes, as well as the inferior RNFL in the right eye. There was also a significant correlation between headache attack duration and the RNFL thickness of the superior retina (p <0.05, Table 6).

**Table 6:** Correlation between frequency of attacks, headache attack duration, duration of migraine, and RNFL thickness in patients with migraine *Spearman’s correlation (ρ); p-value <0.05 is considered significant RNFL: retinal nerve fiber layers; IQR: interquartile range

RNFL thickness	(r) Coef.	*p-value	(r) Coef.	*p-value	(r) Coef.	*p-value
	Frequency of episodes (per month)		Duration of migraine (in years)		Headache attack duration (in hours)	
Superior retina						
Right (median (IQR))	0.07	0.26	0.07	0.03	0.06	0.00
Left (median (IQR))	-0.11	0.10	-0.13	0.001	-0.09	0.00
Inferior retina						
Right (median (IQR))	0.02	0.77	0.11	0.01	0.05	0.17
Left (median (IQR))	-0.04	0.66	-0.09	0.07	0.04	0.43
Nasal retina						
Right (median (IQR))	-0.01	0.97	0.07	0.09	-0.05	0.21
Left (median (IQR))	-0.16	0.22	-0.13	0.05	0.09	0.13
Temporal retina						
Right (median (IQR))	-0.05	0.53	-0.03	0.43	0.07	0.08
Left (median (IQR))	-0.01	0.94	-0.02	0.66	0.001	0.98
Average						
Right (median (IQR))	-0.13	0.50	-0.32	0.004	-0.04	0.60
Left (median (IQR))	-0.27	0.30	0.43	0.002	-0.11	0.37

## Discussion

Migraine is characterized by recurrent episodes of unilateral, pulsating headaches and is usually accompanied by vegetative symptoms such as nausea, vomiting, and excessive sound and light sensitivity [12].

The constriction of cerebral and retrobulbar arteries is linked to the genesis of migraines. Vasospasms and decreased blood perfusion are usually seen in one hemisphere during a migraine attack, while hypoperfusion can also affect other cerebral areas and even the layers of the retina [13]. Reduced flow of blood in the retina and optic nerve may be associated with migraine attacks, resulting in ischemia and irregular ocular perfusion. The recurring vasospasms and focal ischemia that occur during migraine attacks may account for the structural damage to the optic nerve and the ensuing decrease in the thickness of the peripapillary RNFL [14].

Therefore, the monitoring of visual field testing and RNFL thickness is necessary owing to suspected axonal damage in migraine patients [15]. To study ocular impairment and improve our understanding of the pathophysiology of migraine, OCT-based assessments of RNFL may prove to be a valuable method. Thus, in this study, we quantified the variations in RNFL thickness and analyzed its correlation with the clinical presentations of migraine.

We could not find a statistically significant difference in RNFL thickness in all retinal quadrants bilaterally in the patients with migraine compared to controls. No statistically significant differences were found in the RNFL thickness between migraine patients and controls in the study by Gunes et al. [16]. A limited number of migraine attacks or a short mean illness duration may have contributed to these findings.

When comparing patients with migraine to controls in our study, we discovered RNFL thinning in the nasal retina, but this difference was not statistically significant. According to some studies, the nasal quadrant alone had a thin RNFL thickness [8]. The varying susceptibilities of the axons to retinal ischemia were suggested as the cause of the selective involvement of the RNFL. Although few studies pointed towards thinning of RNFL in all quadrants, these results regarding RNFL thickness in migraine patients are inconsistent. Differences in sample sizes and methodologies, as well as ethnic differences, could account for these disparate outcomes.

In our study, we did not find any statistically significant difference in RNFL between patients with and without aura. Similar results were seen by Simsek et al., who found no discernible variation in RNFL thickness across all retinal quadrants [15]. Further, on analysis of the thickness of the RNFL in different quadrants, we found that migraine patients with aura had a thinner temporal RNFL as compared to patients without aura. However, the difference between these two groups was non-significant.

A few studies corroborate our findings. In a study done by Yurtoğullari et al., migraine patients with aura had temporal and inferotemporal peripapillary RNFL quadrant thickness reductions [17]. Significantly less RNFL thickness was observed in the temporal quadrant by Martinez et al. [1].

The pathophysiology of the greater RNFL thinning in migraine with aura patients as opposed to migraine without aura patients is thought to be related to cerebral hypoperfusion in the posterior cerebral hemisphere during migraine aura.

Since several variables may be studied in migraine patients, we looked at the association between RNFL thickness and headache frequency, attack duration, and disease duration. We found a statistically significant correlation between superior and average RNFL thickness and the duration of disease. There was also a significant correlation between superior RNFL and the duration of the headache attack. According to a study by Gunes et al., there is a significant correlation between the length of the headache episode and the superior RNFL thickness, as well as between the frequency of the migraine and the temporal RNFL thickness, which is similar to our results [16].

The RNFL and GCL thickness may be more damaged in migraine patients with a long duration of disease and frequent migraine attacks. Abdellatif and Fouad, in their study, found that there was a negative correlation between superior and inferior RNFL and the duration of migraine [18].

The temporal RNFL thickness was found to have a negative correlation with the frequency of headaches, the duration of migraines, and the MIDAS, according to a study conducted by Martinez et al. [1]. Further clarification was provided by Feng et al., who said that a prolonged migraine duration of more than 15 years can cause a significant reduction in the mean value of RNFL thickness [19].

Although the correlation between RNFL and disability assessed by MIDAS was not significant, there was a negative correlation between these two. That means disability caused by migraine has a negative effect on RNFL thickness. The result is supported by a study done by Martinez et al., who reported a statistically significant correlation between RNFL thickness, MIDAS, and the duration of migraine disease [1].

In our study, the average value of RNFL thickness in all four quadrants was thinner in chronic migraine patients as compared to episodic migraine patients, but it was not a statistically significant difference. Our results are similar to those of Labib et al., in whose study the RNFL thickness of the superior, inferior, nasal, and temporal quadrants, as well as the average RNFL thickness, were substantially lower in chronic migraine patients compared to controls [20].

The comparatively small sample size and the absence of OCT angiography-based vascular evaluation of the retina and choroid at our center were the major limitations of this study.

## Conclusions

The RNFL thickness in migraine patients has been the subject of conflicting research findings. Even if there is a statistically significant reduction in RNFL thickness, the involved quadrants differ across various studies. In our study, we found that the length of migraine disease and the duration of headache episodes have a statistically significant correlation with superior RNFL. There was no significant change in RNFL thickness in our study with respect to other migraine characteristics. Therefore, a multicentric study employing a larger number of patients can be undertaken to reach a conclusion regarding changes in RNFL with migraine variables.
